# HepCare Plus: Enhancing Primary Care Identification and Treatment of Hepatitis C Virus in High-Risk Individuals

**DOI:** 10.3390/pathogens11121428

**Published:** 2022-11-27

**Authors:** Tessa O’Gorman, John S. Lambert, Tina McHugh, Walter Cullen, Gordana Avramovic, Raffaele Federico, Bernard West, Brendan O’Kelly, Louise Vidal, Jeremy Farrell, John Broughan, Eileen O’Connor, James Woo

**Affiliations:** 1 Mater Misericordiae University Hospital, 7 Dublin, Ireland; 2 School of Medicine, University College Dublin, 4 Dublin, Ireland

**Keywords:** hepatitis C virus (HCV), HepCare, peer support, PWID, homeless, linkage to care

## Abstract

Hepatitis C Virus (HCV) disproportionately affects people who inject drugs, migrants, prisoners and the homeless. An integrated, peer-led model of care involving primary and secondary care is required to enhance the identification and treatment of HCV in these marginalised groups. HepCare Plus builds on the network and achievements of HepCare Europe (a co-funded Third Health Programme of the European Union/Health Service Executive project). It further identifies those not accessing care and facilitates prompt assessment and treatment of those diagnosed with HCV, with the aid of a peer support worker (PSW) and a community HCV nurse specialist. Of 109 individuals identified and assessed for HCV treatment, 100 commenced HCV treatment. Despite interruptions to treatment (COVID-19 pandemic and national health service cyberattack) there was a high-level of treatment completion with PSW engagement (98%, n = 98). Eighty (73%) individuals were previously aware of a positive HCV status, highlighting the ongoing need to address barriers preventing marginalised groups from engaging with care. HepCare Plus reiterates the defining role of peer-led community interventions in HCV treatment engagement and the need for continuous open-ended HCV care. It provides a sustainable framework to meaningfully combat HCV and achieve the United Nations Sustainable Development Goal of HCV elimination by 2030.

## 1. Introduction

In the European Union/European Economic area and the United Kingdom (UK) there are an estimated 3.9 million people living with chronic hepatitis C virus (HCV) [1]. However, only an estimated 27% of those living with chronic HCV have been diagnosed [1]. The majority of undiagnosed cases are among high risk populations such as people who inject drugs, migrants, prisoners and the homeless [1]. The 2030 United Nations (UN) Sustainable Developmental Goals (SDG) outline HCV elimination within goal 3, to achieve this, it is essential to target these key populations that are disproportionately affected by HCV [2].

**Figure 1:**
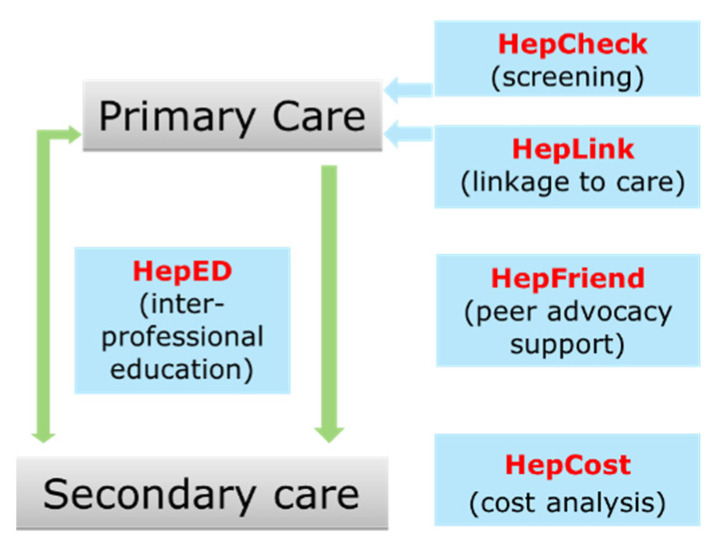
HepCare Europe Model [4].

HepCare Europe was a co-funded Third Health Programme of the European Union /Health Service Executive (HSE) project conducted from 2016 to 2019 [3]. It provides an integrated model of care designed to enhance the identification and treatment of HCV particularly in vulnerable, high risk groups [4]. The model of care involves participation from both primary and secondary care to screen, educate, treat, and support those with difficulty accessing care (Figure 1) [4]. HepCare Europe was initially implemented at four sites across Europe (Ireland, Romania, Spain, UK), including the Mater Misericordiae University Hospital (MMUH) in Dublin, Ireland where links were established between speciality care services providing treatment for HCV (Infectious Disease and Hepatology) and a network of community services in primary care (general practices, harm reduction and homeless primary care services, etc.) [5].

**Figure 2:**
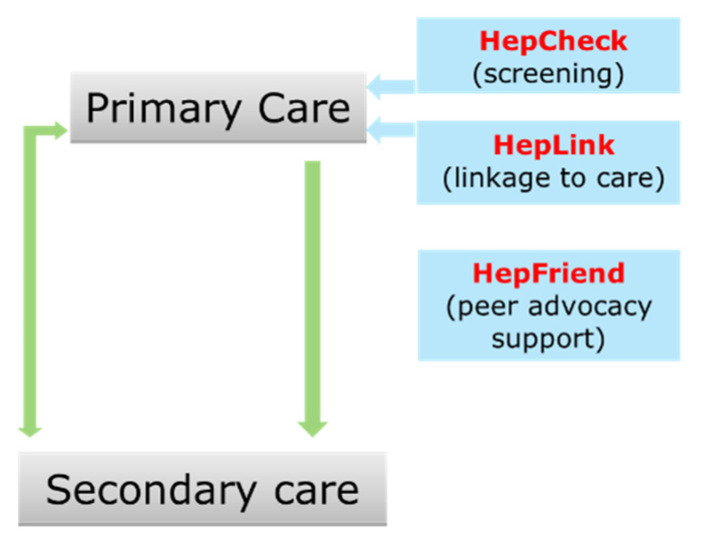
HepCare Plus Model.

The catchment area for the MMUH includes north inner-city Dublin which contains some of Ireland’s most socio-economically deprived areas with populations at high risk of acquiring HCV [6]. Crucially, HepCare Europe identified the need for flexible treatment options beyond the traditional health care structure and the need for additional, tailored supports to firstly engage and then retain individuals on the HCV cascade of care [7]. HepCare Plus is a continuation of the HepCheck, HepFriend and HepLink components of HepCare Europe. (Figure 2) [4].

### Objectives

HepCare Plus aims to build on the networks and achievements of the HepCare Europe model, to further identify those not accessing care, and to facilitate prompt assessment and treatment of those diagnosed with HCV, with the aid of a peer support worker (PSW) and a community hepatitis C nurse specialist. Specifically, HepCare Plus aimed to commence an additional 100 individuals on HCV antiviral treatment over a 12-month period, who may not have otherwise been identified and/or engaged with treatment services. This paper aims to describe components of HepCare Plus and the latest treatment figures in comparison to local and national figures as well as discussing some of the barriers faced, including the extension of the project beyond the anticipated timeline secondary to the COVID-19 pandemic and HSE cyberattack. 

## 2. Methods

### 2.1. Study Design

This is a retrospective descriptive analysis of HepCare Plus, a service enhancement project to strengthen and expand the HepCare Europe integrated model of care for HCV at the MMUH. To be eligible for treatment within the remit of HepCare Plus individuals must have been over 18 years of age, HCV RNA positive, suitable to commence HCV treatment and willing to avail of peer support. 

### 2.2. Access and Engagement

HepCare Plus referrals were received from several sources including:

- Peer facilitated community referrals
- Community addiction services
- General practices
- Homeless services
- Hospital referrals (inpatient and outpatient)

In addition to engaging HCV positive individuals referred from networks established during HepCare Europe, the PSW and community HCV nurse specialist also established new referral links with primary care services. An additional 10 general practices and 22 community services (harm reduction and homeless services) were targeted by the PSW, and nurse specialist as outlined below. 

#### 2.2.1. Peer Led Community Screening Initiatives

On a weekly basis, the PSW attended community-based services where individuals with HCV risk factors also attend, e.g., harm reduction centres and homeless services. At these locations, service users were offered peer facilitated screening, provided with up-to-date information with respect to the transmission, testing and treatment of HCV, and where appropriate offered a direct pathway to treatment. The PSW offered HCV screening depending on individual risk factors via an immediate point of care (POC) OraQuick HCV Rapid Antibody test or referral to a general practitioner (GP)/hospital for HCV serology. For those who tested HCV RNA positive a specialist follow-up appointment was offered as soon as possible with the support of the peer worker.

#### 2.2.2. Direct GP Referral through the Community HCV Nurse Specialist

The community HCV nurse specialist contacted 10 local general practices that had not previously been engaged with during the HepCare Europe project. The nurse provided information regarding the screening process and streamlined access to the HCV cascade of care with the support of a PSW. This enabled GPs to promptly refer any suitable HCV RNA positive individuals directly to the HCV nurse specialist for review. The formation of this community link between general practices and the specialist HCV clinics is crucial in ensuring ease of access to care for this vulnerable cohort.

### 2.3. Peer Support and Linkage to Care

Peer support models of care are conventionally used in mental health services, however recently their role has expanded to other health care services including infectious diseases [8,9]. A PSW has a lived understanding of specific issues affecting the target population, such as a lived experience or recovery from a medical condition and/or lifestyle (e.g., homelessness, substance abuse) [10]. They are also cognisant of the difficulties, inequalities and discrimination faced accessing healthcare. A PSW creates a relatable point of contact for vulnerable individuals, breaking down barriers to promoting health and seeking health services [11]. A PSW can support individual engagement and retention along the HCV cascade of care. A PSW can then empower, facilitate care and advocate for members of the community whilst having the support of a trained nurse specialist. HepCare Europe helped to redefine the role of PSWs in HCV treatment [12]. There are different models for PSWs including volunteers and paid positions. For HepCare Plus the PSW held a paid position based at the MMUH, was specially trained, and at an advanced HCV recovery stage. 

All participants described in the analysis availed of peer support of various levels depending on specific circumstances. The PSW had a lived experience of HCV and previously navigated through some of the services involved. From this experience they had an established network and in-depth knowledge ready to impart on participants. The PSW provided information on the aetiology, diagnosis, and management pathway for HCV in the community. The PSW regularly followed up with patients, reminding them of appointments, referring individuals for specialist care, accompanying them to clinic and providing post-consultation support. The PSW worked closely with community-based key workers. Often the PSW role extended beyond the remit of HCV screening and treatment and included directing individuals to other services such as harm reduction services and social services.

### 2.4. Data Collection and Analysis

All referral agents were requested to submit a dedicated referral form. Additional data was collected retrospectively by chart review and from electronic patient records. Data was analysed using descriptive statistics to evaluate the study population demographics, the cascade of care and compare the findings with overall MMUH and national HCV treatment figures. 

### 2.5. Ethics

Ethical approval was granted by the MMUH Research and Ethics Committee in Dublin REF 1/378/1722, as part of the HepCare Europe programme.

## 3. Results

### 3.1. Demographics 

A total of 109 individuals identified as HCV RNA positive as part of the HepCare Plus project between August 2019 and December 2021 were included in the analysis. The majority, 76% (n = 83) of individuals were male. The median age was 43 years, (IQR 39–48). Twenty-six (23.9%) referrals were received from general practices, 25 (22.9%) referrals were facilitated directly by the PSW, 23 (21.1%) referrals were received from within the hospital setting (inpatient and outpatient), 22 (20.2%) referrals were received from homeless services and 13 (11.9%) referrals were from addiction services. Five (4.6%) individuals were co-infected with HIV (Table 1). Hepatitis B virus (HBV) serology was available for 91 individuals, there were no co-infections with HBV, while 9 (9.89%) individuals had evidence of resolved hepatitis B infection. Data was available on the previous awareness of HCV positive status for 94 individuals, of which 80 (85.1%) individuals were aware of their positive HCV status for more than one year prior to engagement with HepCare Plus. 

### 3.2. Cascade of Care

Of 109 individuals identified as HCV RNA positive, only two were screened using the oral HCV point of care test. 95 (87.2%) individuals were assessed in the Infectious Disease clinic. At the time of writing, 100 (91.7%) of the 109 individuals assessed for treatment had commenced treatment, five individuals were lost to follow up, three individuals are due to start treatment in the coming weeks and one individual’s treatment was deferred. The first participant attended for HCV treatment assessment on 26 August 2019 and treatment was initiated, three weeks later, on the 20 September 2019. The last participant was assessed for treatment on 17 December 2020 and the last participant commenced treatment on the 3 March 2022. The average length of time from assessment to treatment initiation was 5 weeks (range 0–19 weeks). At the time of writing, of the 100 individuals who completed treatment, 68 (68%) had achieved a sustained virologic response (SVR), 30 (30%) completed treatment but did not attend for SVR assessment and two individuals’ treatment defaulted (Table 2). The average length of time from treatment assessment to completion of treatment was 13 weeks (range 8–28 weeks). 

### 3.3. HepCare plus HCV Treatment Figures Compared to Total MMUH and National Figures

Nationally 1598 individuals were commenced on HCV treatment during the HepCare Plus timeframe from September 2019 to March 2022 (Table 3). 17.1% (n = 273) of these individuals were initiated on treatment for HCV in the MMUH. 100 (36.6%) of those who initiated HCV treatment in the MMUH availed of peer support as part of the HepCare Plus project. 

## 4. Discussion

### 4.1. Key Findings

This descriptive report of a population at significant risk of acquiring HCV but low prospect of engaging in HCV screening and treatment found a high-level (98%) of treatment completion with peer support engagement. The basic demographics portrayed are in keeping with the latest annual Health Protection Surveillance Centre (HPSC) Hepatitis C report [13]. The 2017 National screening guidelines for HCV recommend considering birth cohort screening for those born between 1965 and 1985 (currently 37 to 57 years of age) [14]. The number of individuals born within this timeframe consisted of 88% (n = 96) of our population. 4.6% (n = 5) of individuals were already known to be co-infected with HIV. There was no HBV co-infection identified in 91 individuals where HBV serology was available. Of these 9.89% (n = 9) of individuals had evidence of resolved HBV infection. The presence of co-infection with other blood-borne viruses (BBVs) highlights the importance of continually screening for BBVs in high-risk groups and encouraging engagement with harm reduction services to reduce the risk of acquiring other BBVs. Over one third of individuals treated for HCV in the MMUH availed of HepCare Plus instigated peer support. The Infectious Disease and Hepatology teams in the MMUH accounted for 17.1% of individuals treated nationally during this timeframe. Importantly, despite brief interruptions to treatment figures secondary to the COVID-19 pandemic and the HSE Cyberattack, the HepCare Plus model of care proved to be a sustainable pathway for the provision of care to high risk, vulnerable individuals infected with HCV. Overall, the outcomes of HepCare Plus are positive, but a relatively high proportion of individuals did not return for SVR confirmation despite completing treatment. Interestingly, we found that a considerable number of those who engaged with the PSW had prior knowledge of their HCV status. Along with the stigma associated with HCV, another common barrier to engaging with treatment is the misperceived side effects and effectiveness of older HCV treatments [13]. A key role of the PSW and the community nurse specialist included increasing awareness regarding the improved tolerability, effectiveness, and availability of the newer direct-acting antivirals. This highlights the importance of a continuous, open-ended pathway to accessing HCV information, screening, and treatment.

### 4.2. Barriers and Challenges

#### 4.2.1. COVID-19 Pandemic

The COVID-19 pandemic has and will continue to create many challenges and hinder the provision of healthcare services, including HCV screening and treatment. These disruptions are reflected in a reduction in treatment numbers at the MMUH in early 2020 that disproportionately impacted HCV high risk groups [14]. The current uncertain projection of the pandemic reiterates the importance of establishing sustainable community-based HCV management. Notably for HepCare Plus, the provision of peer led care through a dedicated weekly clinic was suspended. Additionally, national public health restrictions limited the access the PSW had to reach, already difficult to reach individuals at high risk of acquiring HCV. The capacity of HCV information sessions/screening was reduced from up to 30 people attending to a maximum of six in attendance. To combat this reduction in attendance, the PSW often relied on word of mouth and distribution of information leaflets to sites where a high proportion of individuals with HCV risk factors frequent. Furthermore, for periods throughout the pandemic, community phlebotomy services ceased. Thus, individuals were required to attend a hospital or general practice setting. This often interrupted and/or delayed therapy or even resulted in the individual being lost to follow up. In contrast, some studies allude to the increased opportunity for diagnosis of chronic HCV and other blood borne viruses amongst key vulnerable populations during COVID-19 pandemic due to the overlapping health disparity of COVID-19 and HCV, providing an opportunity to screen for HCV in hospitalised COVID-19 patients [15]. In keeping with this position, our analysis showed that the hospital setting accounted for 23 (21.1%) referrals for treatment assessment. 

#### 4.2.2. Disruption of HSE Cyberattack

In May 2021, the HSE was subjected to an unprecedented cyberattack. This led to a temporary disruption of many computer-based health services in Irish hospitals. In the MMUH, all new starts for HCV treatment were postponed as it was not possible to register patients via IT systems to the National Hepatitis C Treatment Programme (NHCTP). Thus, undoubtedly affecting HCV treatment figures during this period.

#### 4.2.3. Reluctance of Individuals to Attend the Hospital Setting

Often while individuals were willing to engage in the HCV cascade of care in the community, it proved more difficult to encourage them to attend for necessary follow-up in the hospital setting. In these scenarios the role of the PSW is key to breaking down the barriers to accessing care. Marginalised individuals most at risk for HCV are often reluctant to attend hospitals for various warranted reasons. This was particularly evident with the disruption of the community provision of services during the COVID-19 pandemic, further highlighting the need for improving the community-based services available to this vulnerable cohort. As mentioned previously, a significant portion of individuals were aware of a previous HCV positive status prior to screening and assessment for HepCare Plus. 

### 4.3. Strengths, Limitations and Areas for Future Research

The major strength of this model of care is the inclusion of marginalised groups who are disproportionately affected by HCV. Often, this cohort of people, despite being those with the greatest need are not captured in traditional health screening methods and treatment pathways. This model of care demonstrates both the ability of a PSW to identify those at risk of acquiring HCV and the subsequent ability to retain individuals on the HCV cascade of care. Furthermore, HepCare Plus is likely to be cost effective based on previous evaluations from Ireland and the UK which indicate the potential cost effectiveness of community-based case screening, followed by linkage to care [16,17]. In the UK an intervention that delivered two components of HepCare Europe: HepCheck and HepFriend (Figure 1) indicated that the model improves treatment uptake and could be highly cost-effective. [17] Similarly, in Ireland, the HepLink component evaluated in patients receiving opioid substitution treatment in general practices appears to improve HCV outcomes and be cost-effective [16].

A limitation of this report is the lack of a comparator population and the fact that not all those who engaged with the PSW granted permission for their data to be used. Areas for future research could include a direct comparison to HCV treatment outcomes for those that did not avail of peer support. Additionally, a qualitative study to ascertain why despite being aware of their positive HCV status individuals had not previously engaged with services and importantly to explore what motivated them to do so on this occasion. Furthermore, additional studies exploring the longer-term effects of the disruption caused by the COVID-19 pandemic to the provision of HCV screening and care are required.

## 5. Conclusions

This report reiterates the defining role of HepCare Europe’s peer led interventions in HCV treatment which has reformed the delivery of HCV care to vulnerable, high risk and particularly hard to reach individuals. HepCare Plus enhances the integration of primary and secondary care facilitated by a PSW and a community nurse specialist. It provides a sustainable framework to meaningfully combat HCV and achieve the UN SDG goal of HCV elimination by 2030 [18]. In doing so it offers the right community based care to individuals at greatest risk of acquiring HCV in the right place, at the right time, ensuring not to leave anyone behind [19].

## Figures and Tables

**Table 1:** Demographics of HepCare Plus Hepatitis C virus (HCV) RNA Positive Individuals.

	% (n) (N = 109)
Gender	
⮚Male	76% (n = 83)
⮚Female	24% (n = 26)
Age, years (IQR)	
⮚Median age	43 (39–48)
Co-infection with HIV	4.6% (n = 5)
Aware of positive HCV status >1 year prior to screening *	85.1% (80) *
Referral Source	
⮚General Practices	23.9% (n = 26)
⮚Peer Facilitated	22.9% (n = 25)
⮚Hospital Referral (Inpatient/Outpatient)	21.1% (n = 23)
⮚Homeless Services	20.2% (n = 22)
⮚Addiction Services	11.9% (n = 13)
Treating Service at Mater Misericordiae University Hospital	
⮚Infectious Disease Clinic	87.2% (n = 95)
⮚Liver Centre	12.8% (n = 14)

* Data only available for 94 individuals.

**Table 2:** Cascade of care results.

	Number of Individuals (%)
Of those who were assessed for treatment (n = 109)	
⮚Initiated treatment as of 3 November 2022	100 (91.7%)
⮚Due to commence treatment	3 (2.8%)
⮚Treatment deferred	1 (0.9%)
⮚Lost to follow up	5 (4.6%)
Of those who initiated treatment (n = 100)	
⮚Completed treatment with SVR * as of 3 November 2022	68 (68%)
⮚Completed treatment with no follow up SVR	30 (30%)
⮚Defaulted treatment	2 (2%)

* Sustained Virologic Response (SVR).

**Table 3:** Comparison of Hepatitis C Virus (HCV) treatment figures.

Year	HepCare Plus	MMUH Total	National Figures
2022 *	13	22	133
2021	43	80	554
2020	24	92	567
2019 **	20	79	344
Total	100	273	1598

* To March 2022 ** From September 2019.

## Data Availability

Restrictions apply to the availability of data. Data for National Hepatitis C treatment figures was obtained from the Irish HCV treatment registry and are available on request.
